# A neuroevolution-driven agent-based model of coral larvae settlement

**DOI:** 10.1371/journal.pone.0359316

**Published:** 2026-09-25

**Authors:** Sarah Wolpold, Christian R. Voolstra, Sebastian von Mammen

**Affiliations:** 1 Department of Computer Science, Julius-Maximilians University, Würzburg, Germany; 2 Department of Biology, University of Konstanz, Konstanz, Germany; Bigelow Laboratory for Ocean Sciences, UNITED STATES OF AMERICA

## Abstract

Coral reefs face significant threats due to climate change and human activities, which require innovative approaches to study and protect these ecosystems. Successful settlement is the bottleneck linking a reef’s free-swimming larvae to the benthic community, ultimately determining the next generation of corals and the long-term resilience of the entire ecosystem. This work introduces an agent-based model (ABM) driven by neuroevolution (NE) to simulate coral larval settlement behavior under various environmental conditions. Inspired by biological processes, the model combines sensory input with a neural network (NN) to guide larval actions, optimizing settlement success through evolutionary algorithms. The model replicates three experimental setups from previous studies, validating it against key metrics such as settlement success, vertical distribution, and orientation to environmental cues. The evolved controllers capture the characteristic cue responses of each experiment: crustose coralline algae (CCA)-driven settlement, the bimodal vertical distribution, and orientation towards reef sound, while also exposing the limits of the simplified environment, as its reduced hydrodynamics. Sensitivity analyses further indicate that these behaviors are driven by different mechanisms across the experiments, illustrating how the model yields interpretable, testable outcomes. This approach provides a basis for integrating adaptive behaviors into coral larval simulations, and future work will focus on refining the model to enhance biological realism and scalability.

## 1. Introduction

Corals begin their lives through asexual or sexual reproduction [1]. Although asexually produced corals generally colonize their natal reefs [2], the spawn of sexually produced corals can migrate tens of kilometers through the ocean to settle in a suitable habitat [3–5]. During this journey, the larvae are exposed to a variety of natural hazards, from strong currents to predators [6]. Anthropogenic changes exacerbate stress for coral larvae, as factors such as sedimentation, light pollution and ocean warming can affect their perception and disrupt critical settlement signals [6]. These disruptions not only threaten the survival of coral larvae but also undermine the natural replenishment of reefs, necessitating active interventions to restore coral populations [7].

To counteract coral dieback, various reef restoration programs are exploring solutions such as coral gardening activities [8], substratum enhancement techniques [9], and direct transplantation [10]. In addition to these in-situ measures, computational modeling is used for complementary and predictive purposes [11]. As the movement of coral larvae is strongly dependent on ocean dynamics, most models simulate them with the help of data-based hydrodynamic models [12], resulting in very accurate pathways and connectivity matrices of important source and sink reefs [5]. Considering that the specific larval settlement and decision-making processes do not necessarily change the outcome of these connectivity patterns, they are often simplified in simulations [13]. However, by doing so, current models risk failing to capture the intricate interactions between larvae and their settlement cues, which play a crucial role in shaping spatial patterns of coral settlement [14].

Addressing these limitations, we introduce a NE-driven ABM that maps larval sensory inputs to actions via evolved NNs, enabling adaptive settlement behavior under varying cues. NE is used for this as the cue-to-action mapping itself is uncertain, and the approach allows a single class of evolved controllers to learn that mapping across different experimental contexts and then be tested in altered validation environments without rewriting the behavioral rules by hand. To examine the application, we replicated three well-documented laboratory experiments from the literature in Unreal Engine 5 (UE5), each investigating larval responses to CCA, depth, and sound, respectively. Recreating these, we aimed to test whether the ABM can reproduce key larval responses under controlled conditions, contributing (1) its development; (2) the proof-of-concept replication of the three laboratory experiments on specific settlement cues in a virtual environment, enabling direct comparison with empirical results; and (3) initial evidence that this approach can reproduce larval cue responses, setting the stage for further exploration of how environmental cues govern settlement behavior.

The remainder of this paper first provides an overview of existing coral larval models and how they incorporate settlement dynamics, followed by a description of the proposed model and its implementation. Next, the three laboratory experiments are outlined together with their simulation setups and results. These findings are then discussed in light of their biological relevance and limitations, before concluding with a summary and perspectives for future work.

## 2. Related work

Coral larval dispersal models are predominantly of biophysical nature combined with Lagrangian particle tracking (LPT) that focus on simulating larval movement by tracking particles, i.e., larvae, on their way through the ocean. Each particle is treated as an independent entity whose trajectory is determined by the integration of biological and physical factors [3,12,15]. Thus, LPT models excel at simulating large-scale dispersal patterns, yet their high computational demands and sensitivity to input data quality limit their ability to capture the fine-scale adaptive behaviors of coral larvae in response to dynamic environmental cues [12,15]. Another widely used approach are ABMs, which represent each organism as an agent. In contrast to LPT models, ABMs can include more detailed behavioral dynamics such as the life history, behavior, and interactions of individuals within a population [16]. This allows ABMs to account for variability between individuals, such as differences in swimming abilities, sensory responses and decision-making processes that are critical to predicting settlement success [17]. An example of an ABM focusing on the dynamics of the reef community is the SICCOM model [18]. It includes interactions between coral species and algae, taking into account ecological processes such as growth, competition, and disturbances, with a focus on how different coral and algal characteristics influence the structure of the community. While SICCOM effectively simulates these relationships, it lacks a detailed representation of the sensory-driven behavior of coral larvae, which limits its ability to model early life stages and settlement processes. Similarly, another ABM was presented that effectively models coral community dynamics by integrating trait-based and demographic approaches to simulate the growth, reproduction, and competitive interactions of coral colonies [19]. Although the model provides valuable insights into community-level processes and the role of functional diversity, its design does not readily extend to simulating the sensory-driven behaviors associated with early larval settlement. Concentrating more on early life stages, an ABM was developed to study the connectivity of coral populations in the Arabian/Persian Gulf [20]. This model combines hydrodynamic simulations using the MIKE 3D Flexible Mesh model with an agent-based approach to simulate dispersal and settlement. The model calculates the transport of individual coral larvae by simulating both advection and dispersion using the Langevin equation. The larvae are treated as particles, and biological parameters such as settlement ability, mortality, and vertical swimming behavior were included to evaluate their dispersal routes. However, despite the model being a strong combination of LPT and ABM, the settlement ability is simplified to a process where larvae just settle when there are nearby reefs without any refined decision-making. In contrast to the above approaches, a mechanistic simulation framework was introduced that captures the complex interplay between substrate attractiveness, local hydrodynamics, and spatial configuration in determining coral settlement patterns [14]. A key innovation of this approach is the integration of virtual sampling methods, such as quadrat and settlement tile simulations, which mimic field survey techniques to reveal unexpected settlement patterns and potential sampling biases. This level of detail not only quantifies the impact of fixed environmental parameters on larval distribution but also emphasizes the nuanced effects of substrate arrangement and flow dynamics at fine spatial scales. In this framework, settlement response is prescribed through mechanistic rules, whereas the model proposed in this article lets the response emerge from evolved control, targeting scenarios where the cue-to-action mapping is itself uncertain.

Together, the outlined modeling advances underscore the importance of considering multiple environmental drivers simultaneously. Building on this foundation, evolutionary algorithms, especially Genetic Programming (GP), have increasingly been applied to evolve adaptive strategies in response to environmental cues [21]. The integration of GP-based adaptive behavior with robust mechanistic simulations holds promise for achieving a more comprehensive understanding of coral larval settlement dynamics. GP mimics natural selection by evolving candidate solutions over generations through mechanisms such as selection, mutation, and crossover [22,23]. NE, a specialized form of GP, focuses on evolving NNs [24,25]. In our approach, NE optimizes the NNs that serve as the “brain”, or controller, of individual agents. These networks are composed of input neurons (receiving sensory stimuli from the environment), hidden neurons (highly interconnected processing units), and output neurons (triggering actions such as movement or settlement decisions). Each larva’s NN is encoded in a genome that specifies which neurons are connected and the weights of these connections. Over generations, the genome evolves to optimize behavior in response to environmental conditions. Having demonstrated success in modeling complex ecological phenomena, GP and NE offer a suitable framework for simulating adaptive, sensory-driven behavior in ABMs [26–29].

## 3. Methods

In this section, the proposed NE-based ABM is detailed, including the description of the laboratory experiments that were recreated to investigate and validate this approach.

### 3.1. Model overview

Each larva $a_{i}$ is controlled by an artificial NN that maps environmental inputs to behavioral actions. The networks are evolved through NE, enabling adaptive responses to environmental cues. The model was implemented in Unreal Engine 5.8, adapting the genome-encoded neural-controller representation of D. R. Miller [30] and extending it to a fully three-dimensional (3D) coral-larval-settlement environment.

**ODD-style structure.** Table 1 maps our ABM to the ODD (Overview, Design concepts, Details) protocol [31], a widely used standard for describing ABMs; each element is further mapped to the section that documents it.

**Table 1:** Mapping of our ABM to the seven elements of the ODD protocol [31], with the section documenting each element.

ODD element	Section	Content
Purpose & patterns	Introduction; Sections 3.1 and 3.2	Replication of cue-driven larval behaviors under controlled laboratory-style conditions
Entities, state variables, scales	Section 3.1; Tables 5 and 2	Larval agents, 3D grid cells, environmental cue fields, internal larval states, and experiment-specific spatial setups
Process overview & scheduling	Action mapping; Training and Validation; Section 3.2.2	Sense–process–act cycle per larva, settlement decision, and evolutionary optimization across generations
Design concepts	Section 3.1 (input vector, network dynamics, genome encoding); Section 3.2.2 (validation)	*Sensing*: environmental cue vector ${𝐬}_{i}(t)$; *Adaptation/Learning*: NE of the controller genome; *Objectives*: experiment-specific fitness functions *F*^(*k*)^; *Stochasticity*: random genome initialization, mutation, and random-number-generator (RNG)-decoupled validation seeds; *Observation*: validation metrics with per-run and per-agent logging. *Interaction* and *Collectives*: not represented; larvae do not sense or influence one another
Initialization	Section 3.2.2 (Training and Validation)	Random initialization of larval positions and orientations within experiment-specific release regions
Input data	Sections 3.2.1 and 3.2.2; Tables 5 and 6	Laboratory-inspired geometry and the environmental cue/parameter fields that drive each experiment
Submodels	Section 3.1 (network dynamics, action constraints, competency-gated settlement); Section 3.2.2 (fitness/reward equations)	Sensory-to-action network map $f_{\theta_{i}}$, movement update with bounds, competency-gated settlement, and the experiment-specific fitness/reward definitions

**Fig 1:**
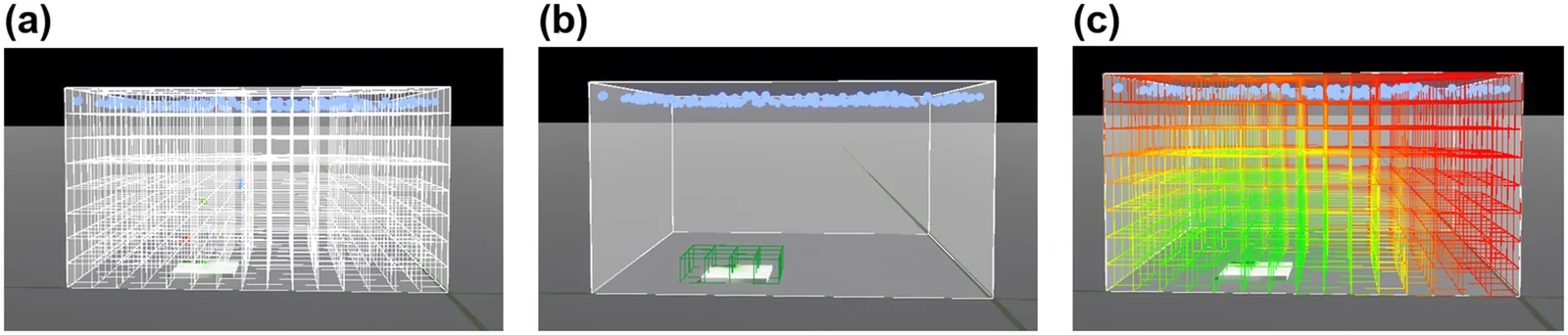
Environmental-grid representation used for E1 [33]: (a) the discretized 3D aquarium domain, (b) the reef-cell subset around the tile, (c) the Gaussian CCA field. (a) Aquarium divided into grid cells $ℰ\subset {𝐑}^{3}$, with larvae (blue spheres) positioned in their respective cells. (b) Grid cells (black) defined as reef cells ℛ⊂ℰ surrounding a CCA-covered tile at the bottom. (c) Distribution of CCA concentration C(𝐱), modeled with a Gaussian decay (red = 0%, green = 100% cover).

**Input vector.** The hydrodynamic environment (see Fig 1) is represented as a 3D grid $ℰ\subset {\mathbb{R}}^{3}$, where each cell 𝐱∈ℰ contains temperature T(𝐱), salinity S(𝐱), pressure Π(𝐱), CCA concentration C(𝐱), a water current specified by its direction $\theta_{c}(𝐱)$ (a unit vector) and scalar magnitude $F_{c}(𝐱)$, and light intensity $I_{\ell }(𝐱,\lambda )$ at wavelength λ. The acoustic cue is represented as a scalar particle-motion field $u_{p}(𝐱)$ derived from the source level, under a plane-wave assumption. For each cell the sound pressure level (SPL) summed over the sources is


$$\begin{array}{c} SPL(𝐱)=L_{0}-11.1\;\log_{10}d(𝐱), \end{array}$$

with source level *L*_0_ = 153 dB re 1μPa at 1 m and distance d(𝐱) to the source; this is converted to a pressure p(𝐱) and then to a particle-motion magnitude $u_{p}$ proportional to $p/(\rho c\;2\pi \bar{f})$, evaluated at a representative frequency $\bar{f}$ (the midpoint of the 100–10,000 Hz band), where ρ is seawater density and *c* the sound speed. This scalar field is a simplified, distance-attenuated proxy for the acoustic cue; directional information reaches the controller as spatial gradients of $u_{p}$ across neighboring cells. The current direction $\theta_{c}(𝐱)$ and magnitude $F_{c}(𝐱)$ influence movement through the local water field. When a low-intensity current is enabled (E1 scenario V1D, E2 scenario V2D, E3 scenario V3D), its direction is a fixed horizontal unit vector ($\theta_{c}=(0,1,0)$, constant in space and time) and its magnitude is a dimensionless depth-decreasing factor $F_{c}(𝐱)=1-z/z_{\max}\in [0,1]$, strongest at the surface and vanishing at the bottom, with no temporal variability. At this stage of the model, this is thus a qualitative flow perturbation, not a calibrated water velocity in $cm\;s^{-1}$. Specific positions on the grid are designated reef cells (the subset ℛ⊂ℰ) and carry the CCA and *Alteromonas* settlement cues, where *Alteromonas* serves as a representation for a biofilm-associated chemical signal.

At time *t*, larva $a_{i}$ located at ${𝐱}_{i}(t)$ perceives a sensory input vector


$$\begin{array}{c} {𝐬}_{i}(t)=[T_{i},\;\Pi_{i},\;C_{i},\;S_{i},\;u_{p,i},\;I_{\ell ,i},\;A_{i},\;E_{i},\;P_{i}{]}^{T}, \end{array}$$

where each environmental channel is the corresponding field sampled at the larva’s position, i.e., a function of ${𝐱}_{i}(t)$: $T_{i}=T({𝐱}_{i}(t))$, and likewise $S_{i},\Pi_{i},C_{i},u_{p,i}$, and $I_{\ell ,i}$. The remaining three inputs are internal-state variables, not sampled from the field: the larva’s age $A_{i}(t)$ and energy $E_{i}(t)$, and an internally adjustable oscillator period $P_{i}(t)$ that supplies the controller with a periodic signal the other state variables cannot represent.

In our implementation, every larva has access to the full sensor set (chemical, light, acoustic/particle-motion, and internal-state channels); which of these are actually wired to hidden or action nodes is determined by the evolved genome rather than fixed per experiment. Sensors whose field does not vary in a given setup, for example acoustic particle motion in the CCA-only E1 setup, could therefore be pruned or carry negligible weight. The cue-to-behavior mapping is thus an evolved outcome, not a hand-specified wiring.

**Action mapping.** The larva’s NN, parameterized by $\theta_{i}$, maps the input vector to an action:


$$\begin{array}{c} {𝐚}_{i}(t)=f_{\theta_{i}}({𝐬}_{i}(t)), \end{array}$$

where ${𝐚}_{i}(t)$ includes forward strength $U_{i}(t)$, rotation angles (yaw $\Delta \phi_{i}(t)$ and pitch $\Delta \vartheta_{i}(t)$), oscillator adjustments, and a settlement decision. Here, $\phi_{i}(t)$ and $\vartheta_{i}(t)$ denote the larva’s current yaw and pitch orientations, so $\Delta \phi_{i}(t)$ and $\Delta \vartheta_{i}(t)$ are incremental steering updates generated by the controller. Actions are subject to physical and biological constraints, e.g.,


$$\begin{array}{c} 0\leq U_{i}(t)\leq U_{\text{max}},\;|\Delta \phi_{i}(t)|\leq \Delta \phi_{\text{max}}. \end{array}$$

Internal variables such as $A_{i}(t)$, $E_{i}(t)$, and a competency threshold *A*_competency_ further influence movement limits and settlement eligibility. Biologically, *A*_competency_ represents the onset of settlement competence, i.e., the minimum larval age at which attachment to suitable substrate is allowed.

**Network dynamics.** Formally, the network is a directed graph composed of sensor nodes, hidden neurons, and action nodes. The input layer is exactly the sensory vector, $h^{\left(0\right)}={𝐬}_{i}(t)$, and a standard feedforward formulation applies (symbols in Table 2):

**Table 2:** Main symbols and indices used in the model description.

Symbol	Meaning
$a_{i}$	Larval agent *i*
𝐱∈ℰ	Spatial cell position in the 3D environment grid
${𝐱}_{i}(t)$	Position of larva *i* at time *t*
ℛ⊂ℰ	Set of reef cells that permit settlement
${𝐬}_{i}(t)$	Sensory input vector perceived by larva *i* at time *t*
${𝐚}_{i}(t)$	Action vector produced by the NN of larva *i*
$\theta_{i}$	Genome/NN parameterization of larva *i*
$f_{\theta_{i}}$	Controller map induced by the genome $\theta_{i}$ (input vector → action vector)
*g*	Gene (*sType*,*sIdx*,*tType*,*tIdx*,*w*): source/target node type and indexes, connection weight
$h^{\left(l\right)},W^{\left(l\right)},b^{\left(l\right)},\sigma ,L$	Hidden-layer state, weight matrix, bias, activation function, and network depth
$U_{i}(t)$	Forward swimming strength at time *t*
$U_{\text{max}},\Delta \phi_{\text{max}}$	Maximum forward strength and maximum per-step yaw increment
$\phi_{i}(t),\vartheta_{i}(t)$	Current yaw and pitch orientation of larva *i*
$\Delta \phi_{i}(t),\Delta \vartheta_{i}(t)$	Incremental yaw and pitch updates generated by the controller
$F^{\left(k\right)}(\theta_{i})$	Experiment-specific fitness of larva *i*, k∈{E1,E2,E3}
*d*_reef_	Final distance to the nearest reef cell (E1 fitness)
$d_{s},d_{s,0}$	Final and initial distance to the sound-source centroid (E3 fitness)
*z*_0_	Larval release height in the vertical column (E2 fitness)
$u_{p}$	Local scalar particle-motion magnitude (E3 acoustic cue)
$R_{CE}$	Clarke–Evans aggregation index (values <1 indicate aggregation)
T(𝐱),S(𝐱),Π(𝐱)	Temperature, salinity, and pressure at spatial position 𝐱
C(𝐱),B(𝐱)	CCA and *Alteromonas*/biofilm concentration fields at 𝐱
*S*_set_	Terminal settlement score in the E1 fitness
$L_{0},SPL(𝐱)$	Acoustic source level and cell sound pressure level
ρ,c	Seawater density and sound speed in water used in the particle-motion conversion
$I_{\ell }(𝐱,\lambda )$	Light-intensity field at position 𝐱 and wavelength λ
$\theta_{c}(𝐱),{𝐅}_{c}(𝐱)$	Current direction and current-force field at spatial position 𝐱
$A_{i}(t),E_{i}(t),P_{i}(t)$	Age, energy, and oscillator-period state of larva *i*
*A*_competency_	Minimum age at which settlement is permitted


$$\begin{array}{c} h^{\left(l\right)}=\sigma \;(W^{\left(l\right)}h^{\left(l-1\right)}+b^{\left(l\right)}),\;l=1,...,L,\;{𝐚}_{i}(t)=W^{\left(L+1\right)}h^{\left(L\right)}+b^{\left(L+1\right)}, \end{array}$$

where *W*^(*l*)^ and *b*^(*l*)^ are the layer-*l* weight matrix and bias, *h*^(*l*)^ the layer-*l* activations, *L* the number of hidden layers, and σ the hidden-layer activation function. This composition of layers is precisely the controller map ${𝐚}_{i}(t)=f_{\theta_{i}}({𝐬}_{i}(t))$ from the action-mapping equation above; $f_{\theta_{i}}$ is induced by the genome parameters $\theta_{i}$.

**Genome encoding.** Each network is encoded by a genome $\theta_{i}$, which specifies both topology and weights. Each connection is represented by a gene


$$\begin{array}{c} g=(\text{sType},\text{sIdx},\text{tType},\text{tIdx},w), \end{array}$$

where *sType*, *tType* denote source and target node types, indices *sIdx and tIdx*i, respectively, dentify node IDs, and w∈ℝ is the connection weight. Concretely, source nodes can be sensors or hidden neurons, whereas target nodes can be hidden neurons or actions. The encoded sensor identities, therefore, determine which measured cue is connected to which hidden or action node, making the cue-to-behavior mapping explicit at genome level.

**Boundaries, settlement, and geotaxis.** Three further rules complete the model specification. First, the aquarium walls are impermeable: each step a larva’s position is clamped to the domain, and a wall-colliding larva’s heading toward the wall is removed, so it cannot leave the tank. Second, settlement is gated by a per-larva competency age *A*_competency_ (heterogeneous when $A_{\text{competency}}^{\min}<A_{\text{competency}}^{\max}$): before competence a larva cannot settle, and thereafter it settles stochastically each step with probability equal to the settlement output of its controller — a continuous scalar clamped to [0,1] and used directly as the per-step settlement probability, so premature settlement is prevented rather than penalized. Third, once competent, a larva carries a constant downward behavioral geotaxis (0.05cm/step). This bias is additive: each step the fixed downward increment is added to the controller’s own movement and to any water current, and the resulting position is clamped to the tank. Because it is well below the maximum swim step, a controller swimming upward more strongly overrides it. It provides the descent tendency in the depth task; this reflects the ontogenetic shift toward downward swimming and substrate approach at the onset of competence reported for coral larvae [32].

**Training and validation.** The model is used in two phases. In the *training* phase, the controllers are evolved; in the subsequent *validation* phase, the fixed evolved controllers are deployed in modified environments to test whether the learned behavior transfers. During the training phase, genomes are initialized randomly and optimized by the experiment-specific fitness functions $F^{\left(k\right)}(\theta_{i})$ that reward the target behavior of each experiment. Optimization proceeds via evolutionary operators applied to a *population* of genomes across generations: *selection* keeps the higher-fitness genomes as parents (here Elitism, Truncation, or Stochastic Universal Sampling, SUS), *crossover* recombines two parent genomes into an offspring genome, and *mutation* randomly perturbs individual genes (connection weights and, through added/removed genes, the network topology). These operators are governed by parameters such as population size, genome length, and mutation rate. In the subsequent validation phase, evolved networks are evaluated in modified environments to assess robustness. Computations are parallelized across threads for efficiency.

### 3.2. Experiments

Building on the model described above, we present the replication of the three laboratory experiments (E1, E2, E3), each targeting a distinct aspect of larval behavior. These provide the reference points used below to evaluate how closely the model reproduces the observed dynamics.

#### 3.2.1. Original experiments.

Table 3 provides a summary of experimental setups, investigation topics, and key findings of the original laboratory studies on coral larval settlement and behavior, highlighting their respective methodologies and observed actions. Collectively, these demonstrate that coral larvae exhibit distinct, cue-specific responses: they preferentially settle on substrates enriched with biofilms and CCA, shift their vertical distribution over the larval period, and orient towards acoustic signals. These findings provide the behavioral benchmarks against which we tested our model.

**Table 3:** Summary of original experiments and validation setups. Validation-setup letters follow a shared scheme (A: base; B/C: variation of the experiment’s manipulated cue — CCA cover in E1, source position along the tube in E3; D: imposed current; E: no-cue control; F: alternative source geometry), so a letter is present only where its manipulation applies (e.g., E2 varies no such cue and therefore has no B/C).

	Setup	Investigation Topics	Key Observations	Validation Setups
**E1** [33]	CCA and biofilm-covered tiles in aquaria with filtered seawater; larvae released and observed after two days.	Settlement success, nearest-neighbor distance, Clarke–Evans index, and settlement in contact.	Larvae settled on tiles and in contact with each other, moving towards CCA-covered areas.	**V1A**: as in the original **V1B**: reduced CCA cover **V1C**: increased CCA cover **V1D**: imposed current **Patch sweep**: max tile area 5–$57\;cm^{2}$
**E2** [32]	Biofilm-covered tiles placed in vertical tubes; vertical distribution recorded over 16 days under day-night light cycles.	Vertical larval distribution at different depths over the larval period.	Larvae shifted downward over time, producing a bimodal top/bottom distribution.	**V2A**: as in the original **V2D**: imposed current **Light sweep**: attenuation $K_{d}$ over 0.025–$0.4\;m^{-1}$ (see Fig 7)
**E3** [34]	Six tubes arranged around three sound sources playing reef sounds of varying frequencies and intensities.	Vertical and horizontal larval distribution analyzed using chi-square and ANOVA tests.	Larvae gathered in the parts of the tubes closest to the sound source, responding to acoustic signals.	**V3A**: as in the original **V3B/C**: source moved along the tube **V3D**: imposed current **V3E**: no sound **V3F**: source below the tube **Acoustic sweep**: source level 140–160 dB and frequency band (see Fig 8)

#### 3.2.2. Computational experiments – replications.

**Fig 2:**
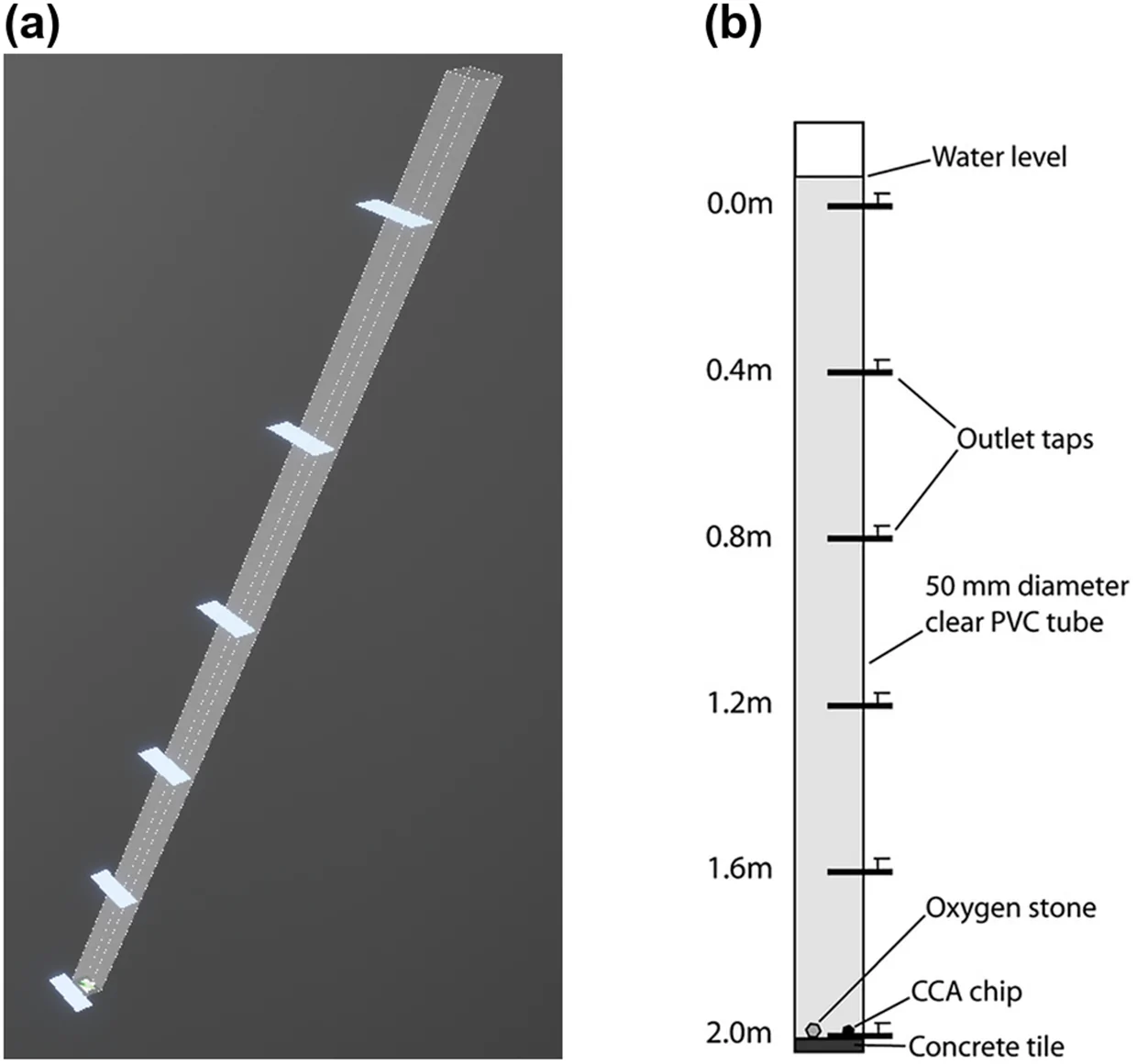
E2 vertical-distribution setup: (a) simulated UE5 geometry and (b) laboratory geometry in [32]. (a) Simulated vertical tube setup in UE5. (b) Laboratory vertical tube setup in [32].

**Fig 3:**
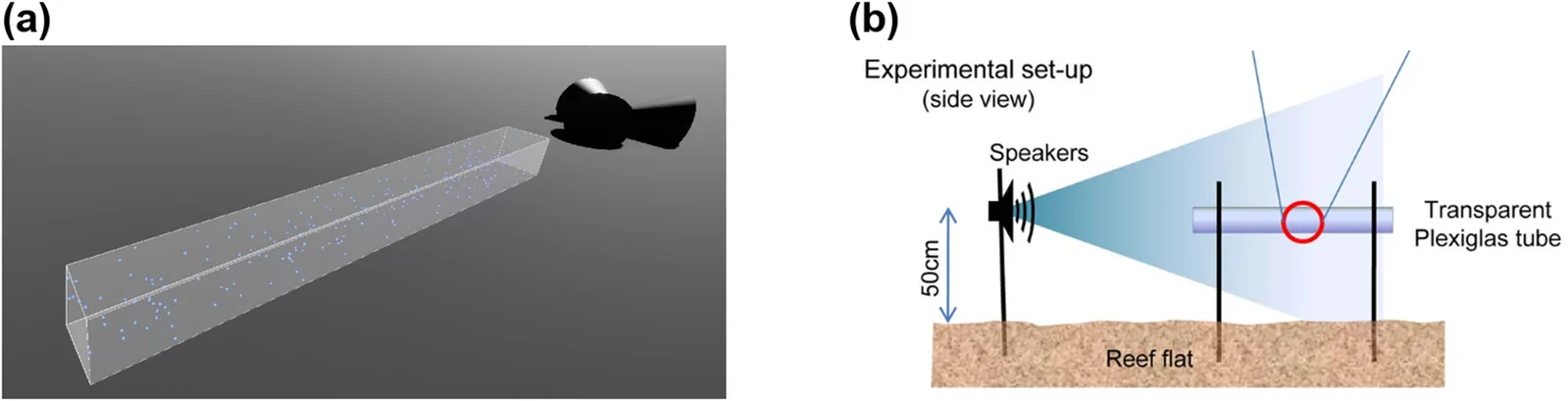
E3 acoustic-choice setup: (a) simulated UE5 geometry and (b) laboratory geometry in [34]. (a) Simulated acoustic-choice setup in UE5. (b) Laboratory acoustic-choice setup in [34].

Accordingly, in the replication with our model (see Figs 1–3), a separate fitness function was defined for each experiment to reward its target behavior. Each function scores a larva $a_{i}$ with controller $\theta_{i}$ by the trajectory it produces over one simulation run.

**E1 (settlement on CCA-covered tiles).** The fitness rewards approaching and settling on the CCA-covered reef:


$$\begin{array}{c} F^{\left(E1\right)}(\theta_{i})=\frac{c_{d}}{d_{\text{reef}}}\;+\;c_{a}\;(C+10\;B)\;\kappa_{\text{reef}}\;+\;S_{\text{set}}, \end{array}$$

where *d*_reef_ is the final distance to the nearest reef cell ($c_{d}=500$); *C* and *B* are the local CCA and *Alteromonas*/biofilm concentrations ($c_{a}=10$); $\kappa_{\text{reef}}=50$ if the larva ends on a reef cell and 1 otherwise; and the terminal settlement score is *S*_set_ = +2500 for settling on a reef cell, −100 for settling off-reef, and −30 for not settling.

**E2 (vertical distribution).** This fitness has no chemical or settlement term; it rewards the competency-gated descent that reproduces the observed vertical migration. With release height *z*_0_ and final height *z*,


$$\begin{array}{c} F^{\left(E2\right)}(\theta_{i})=\left\{ \begin{array}{cc} z_{0}-z & \text{if }A_{i}\geq A_{\text{competency}},\\ {}[2pt]z & \text{otherwise,} \end{array}\right. \end{array}$$

so before competence a larva is rewarded for remaining high in the column, and once competent for descending toward the substrate; settlement itself is score-neutral.

**E3 (phonotaxis).** The fitness rewards approaching the sound-source cluster and residing where the particle-motion cue is strong:


$$\begin{array}{c} F^{\left(E3\right)}(\theta_{i})=c_{1}\;\left(1-\frac{d_{s}}{d_{s,0}}\right)+c_{2}\;u_{p}^{2}+c_{3}\;(d_{s,0}-d_{s}), \end{array}$$

where $d_{s}$ and $d_{s,0}$ are the final and initial distances to the centroid of the sound sources, $u_{p}$ is the local scalar particle-motion magnitude, and $c_{1}=c_{2}=5000$, *c*_3_ = 500. These constants are empirical reward weights, not fitted parameters: because selection uses only the within-generation *ranking* of fitness, their absolute magnitude is immaterial and only their ratio matters. Approaching the source and residing where the cue is strong are weighted equally ($c_{1}=c_{2}$), with the absolute distance-reduction term a smaller, secondary contribution (*c*_3_); the reward weights of the E1 and E2 fitness functions were set on the same empirical, ratio-based basis.

For E2 specifically, the competency-onset window ($A_{\text{competency}}^{\min}$, $A_{\text{competency}}^{\max}$) and the geotactic descent speed were calibrated so that the simulated depth-versus-time course matched the sampling points reported by [32]. In order to train the larval controllers towards the laboratory behaviors, six training rounds with different parameters were carried out to achieve an optimal learning result. Each experiment (E1, E2, E3) was trained *independently*: with its own experiment-specific fitness function and its own set of runs, never mixed or interleaved across experiments. These six training rounds are six *configurations* applied per experiment — three comparing the selection methods and three sweeping hyperparameters (mutation rate, genome length, population size), enumerated in Table 4. The baseline configuration used population size 250, genome length 50, maximum inner-neuron count 12, selection rate 0.1, rest-selection rate 0.5, overlay crossover, and multi-point mutation with rate *p*_mut_ = 0.005, evolved over *G* = 1000 generations. The per-run simulation horizon depended on the experiment: E1 and E3 used 1000 steps, whereas E2 used a longer horizon (5000 steps at baseline, extended to 10,000 in the longest-descent rounds) to accommodate the 2.2 m vertical tube. Movement is not gated by competency — larvae swim from release — with the per-step forward displacement capped at the maximum forward strength ($U_{\max}$, of order 1 cm/step at the model’s 1 UU = 1 cm scale). Over a 10,000-step horizon the reachable path length is therefore on the order of 100 m, roughly two orders of magnitude beyond the 2.2 m column, so the horizon is not limiting for descent and settlement. The six training rounds primarily explored selection strategy, mutation rate, genome length, and population size around this baseline (see Table 4). For each training round, larvae were initialized by uniform random sampling within the release region: in E1 and E2, *x* and *y* positions were sampled uniformly across the surface release area while *z* was fixed at the top layer of the aquarium, whereas in E3 the *x*, *y*, and *z* coordinates were all sampled uniformly across the available 3D release volume. Initial yaw and pitch were likewise sampled uniformly from $0^{\circ }$ to $360^{\circ }$. Trained controllers were then evaluated on the task-specific validation scenarios.

**Table 4:** Training rounds. Rounds R1–R3 compare the three selection methods (Elitism, Truncation, SUS) at the baseline configuration; rounds R4–R6 sweep mutation rate, genome length, and population size using the selected SUS method. Baseline: population 250, genome length 50, 12 hidden neurons, *p*_mut_ = 0.005, 1000 generations.

Round	Primary change	Selection	Pop.	Genome	Other settings
R1	Baseline	Elitism	250	50	*p*_mut_=0.005; 1000 steps in E1/E3; 5000 steps in E2
R2	Selection sweep	Truncation	250	50	*p*_mut_=0.005; 1000 steps in E1/E3; 5000 steps in E2
R3	Selection sweep	SUS	250	50	*p*_mut_=0.005; 1000 steps in E1/E3; 1000–10000 steps in E2
R4	Reduced mutation	SUS	250	50	*p*_mut_ = 0.001
R5	Reduced genome length	SUS	250	25	*p*_mut_ = 0.005
R6	Reduced population size	SUS	125	50	*p*_mut_ = 0.005

The trained controllers were then evaluated in the validation environments, each parameterized to match the geometry and cue ranges of the corresponding experiment (Tables 5 and 6): for E1, a shallow arena with CCA-covered limestone tiles (combined substrate area ≈2–57 cm^2^); for E2, a 2.2 m vertical tube of 50 mm inner diameter; and for E3, a single representative 1 m tube (10 cm diameter) along the source axis (the original’s six radially arranged chambers reduce to one such tube in the simulated 100×10×10 cm grid). Environmental fields followed each original study: E2 additionally imposed the diel 24.0–$33.2{\;}^{\circ }$C temperature cycle and the day–night light cycle, whereas E1 and E3 used a constant temperature and the default salinity of 35 PSU, as their source studies specified no other overrides. In E1, the species-specific fragment counts of the original were abstracted as tiles of variable per-run size and combined cover, so the comparison is anchored to the CCA and substrate-area manipulations, not to a literal reconstruction of fragment counts. Each experiment also included the simplified-current perturbation described above.

**Table 5:** Model parameters used to generate the environmental cue fields.

Parameter	Value	Context
Spatial scale	1 world unit = 1 cm	Physical scale applied to all final experiment grids, meshes, larvae, and movement strengths
Grid cell edge length	1 cm	Cell size (CellEdgeLength=1) used in all final experiment grids
E1 grid bounds	15×15×8 cm	Aquarium domain size used for the final E1 runs
E2 grid bounds	5×5×220 cm	Aquarium domain size used for the final E2 runs
E3 grid bounds	100×10×10 cm	Aquarium domain size used for the final E3 runs
	25–$29{\;}^{\circ }$C	Constant temperature interpolated across the grid
Salinity	35 PSU	Mean salinity assigned across the default water column
Surface pressure	1 atm	Surface pressure used before depth-based hydrostatic increase
Light attenuation (default grid)	0.085	Default attenuation used for general light decay with depth
Depth for blue dominance	10 m	Depth threshold used for wavelength shift toward the blue range
E2 attenuation coefficient $K_{d}$	0.1	Vertical tube light-cycle setup (E2 base); swept over 0.025–0.4 in the light-sensitivity analysis
E2 temperature cycle	24.0–$33.2{\;}^{\circ }$C	Sinusoidal daily forcing with peak at hour 15
E3 source level	153.0 dB re 1μPa at 1 m	Acoustic source level used to derive particle motion
E3 frequency range	100–10000 Hz	Frequency range used in the acoustic field calculation
Sound speed in water	1500 m/s	Constant used in particle-motion conversion
Current direction	(0,1,0)	Direction imposed when the simplified current field is enabled
Current force	$1-z/z_{\text{max}}$	Dimensionless depth-decreasing factor
CCA gradient spread	σ=5	Gaussian spatial spread used for off-reef CCA concentration
*Alteromonas* scaling	0.9	*Alteromonas* concentration set as 0.9×CCA in reef cells

**Table 6:** Original-study parameters informing the validation setups, grouped by experiment.

Setup quantity	Value	Context
**E1: CCA settlement [33]**		
Empirical fragment counts	30, 36, 10	Fragment counts reported for *A. valida*, *A. digitifera*, and *An. spinosa*
Total substrate area	ca. 2–57 cm^2^	Substrate-area range used as the control parameter
Bowl geometry	15×8 cm; ca. 500 mL filtered seawater	Physical container and water volume
Larval age / exposure	3–4 days old; 48 h	Reported larval age and observation duration
**E2: vertical distribution [32]**		
Tube geometry	2.2 m height, 50 mm inner diameter	Physical dimensions of the tube setup
Outlet heights	0.0, 0.4, 0.8, 1.2, 1.6, 2.0 m	Vertical sampling zones
Acclimatization / sampling	16–22 h; 0800–1600 h	Acclimatization and daylight sampling windows
Settlement substrate	concrete tile + CCA chip at tube bottom	Physical settlement target
**E3: phonotaxis [34]**		
Chamber geometry	6 chambers, each 1 m long and 10 cm in diameter	Physical chamber layout
Speaker placement	1 m from chambers, 0.5 m above them	Relative geometry of the setup
Larvae per chamber	500	Reported release size

**Statistical analysis.** Every reported validation value is a mean over all 300 runs of a setup (30 genome seeds × 10 RNG seeds), which sample both controller variation (the genome seeds) and stochastic-decision variation (the RNG seeds). Within a run we do not rely on engine-level determinism; instead, each larva draws its stochastic decisions (e.g., settlement) from a private random stream seeded deterministically from the run seed, the generation, and the agent index, so those outcomes are reproducible and independent of thread scheduling, and the environmental fields are static within a run. The reported means are therefore reproducible functions of the (genome, RNG) seeds rather than of non-deterministic thread order, which is what the 10 RNG seeds per genome are designed to average over. Statistics followed the analysis of each original study, adapted to the multi-seed design.

For E1, we reported the mean percentage of larvae settling on reef cells with standard errors and 95% *t*-based confidence intervals across runs, and quantified spatial clustering from the final settlement coordinates with the Clarke–Evans index $R_{CE}$ ($R_{CE}<1$ indicating aggregation). Following the original settlement–area model, we fitted a binomial (logit) generalized linear model (GLM) of correct settlement on substrate area; because area is constant within a scenario, the slope was estimated across the patch-size sweep (maximum tile areas 5/15/30/$57\;cm^{2}$), with a companion model using CCA cover across V1B/V1A/V1C, both with a quasibinomial link to absorb within-run overdispersion (≈250 non-independent larvae per run). The original’s larva–larva contact model was not fitted, as the present model has no larva–larva sensing.

For E2, we summarized the vertical distribution as the fraction of larvae in each of six depth zones and compared the simulated depth profile against the profile reported by [32].

For E3, horizontal occupancy was binned into five source-distance zones and vertical occupancy into five height zones. A $\chi^{2}$-vs-uniform statistic and a one-way ANOVA (*F*_4,1495_, *n* = 300 runs) described non-uniformity, but because the ≈75,000 pooled larvae per setup are non-independent (larvae within a run share one controller and environment) these were treated as descriptive only. The primary, pre-specified test of phonotaxis was the source-tracking of the mean horizontal position at the independent-seed level (*n* = 30 genome seeds): a paired Wilcoxon signed-rank test of per-seed mean *x* between the source-near (V3C) and source-far (V3B) placements, and between the sound-on (V3A) and silent (V3E) controls.

## 4. Results

We first describe the training progression and optimization behavior, then the validation results for E1–E3.

Across the successful training runs, the learning trajectory followed a consistent pattern. In the initial generations, controllers moved largely at random and rarely reached the reward-bearing regions, yielding low fitness. Over successive generations they learned to approach the relevant cue: the CCA-covered tile in E1, the lower part of the column in E2, and the sound source in E3, as well as, in E1, to settle on the reef cells once competent.

**Selection method and hyperparameters.** Selection methods and hyperparameters were ranked on each experiment’s primary biological-outcome metric, defined under *Statistical analysis* above (E1 correct-settlement rate; E2 depth-profile match to the Tay time course; E3 source-tracking of the mean horizontal position). On this metric, SUS was selected in all three experiments (Friedman omnibus across the three methods, *df* = 2; Holm-corrected pairwise Wilcoxon signed-rank post-hoc tests; Kendall’s *W*, *N* = 30 seeds): E1 $\chi^{2}=43.3$, *p* < 0.001, *W* = 0.72 (large); E2 $\chi^{2}=9.9$, *p* = 0.007, *W* = 0.16 (small); E3 $\chi^{2}=7.4$, *p* = 0.025, *W* = 0.12 (small); all pairwise comparisons were Holm-significant. The hyperparameter effects (four-configuration Friedman, *df* = 3: E1 $\chi^{2}=30.6$, E2 $\chi^{2}=45.9$, E3 $\chi^{2}=38.7$, all *p* < 0.001; *W* = 0.34/0.51/0.43) were task-dependent: a reduced genome length lowered E1 settlement but improved E3 phonotaxis, and a reduced population gave the best E2 depth match.

**Fig 4:**
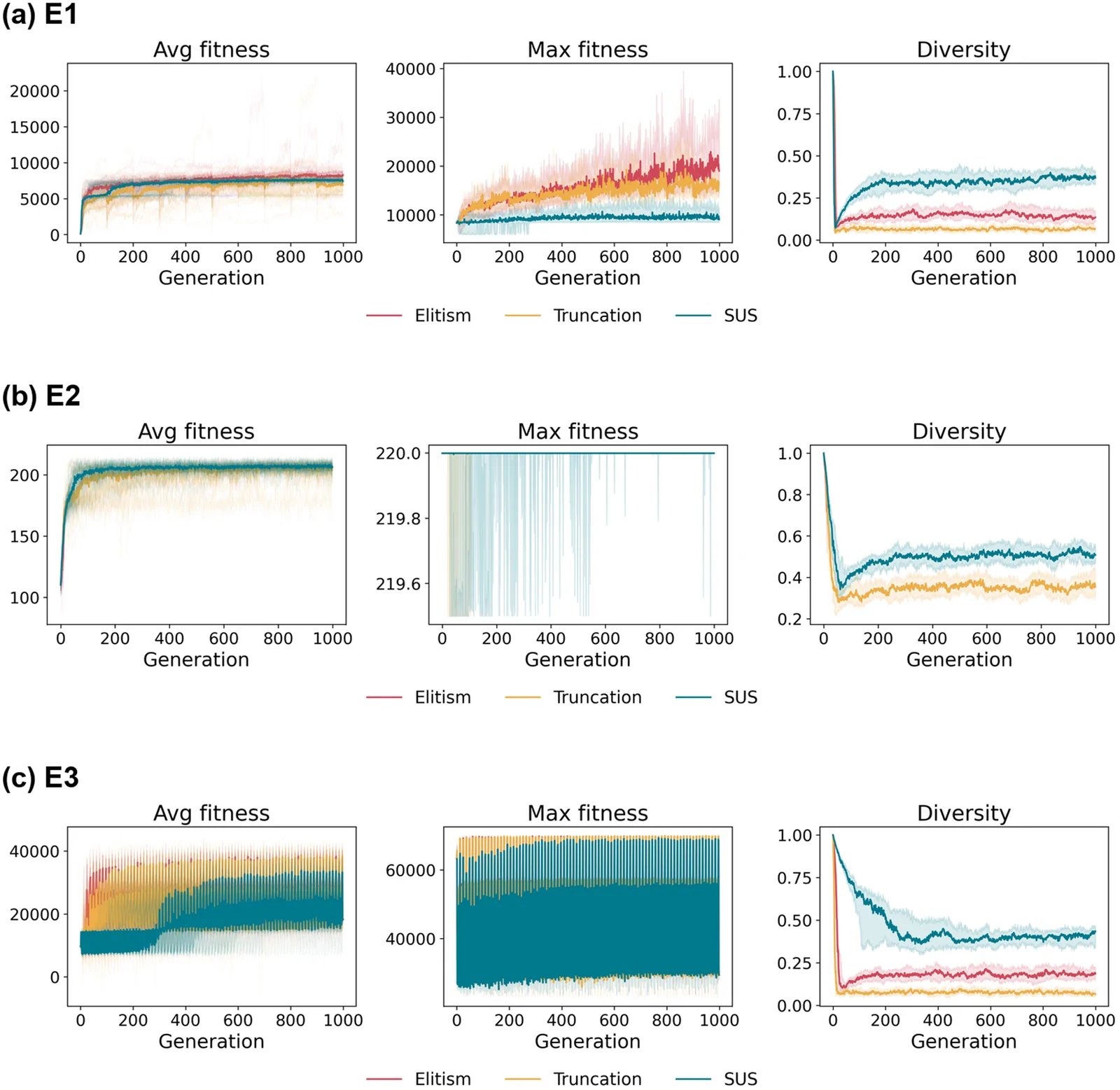
Genetic-algorithm convergence and reliability across the 30 seeds of each experiment: population mean fitness, maximum fitness, and genetic diversity versus generation. Bold lines are the across-seed median; faint lines are individual seeds. All seeds improve over the generation-0 baseline and diversity declines smoothly. (a) E1. (b) E2. (c) E3.

**Convergence and reliability.** Across all three experiments and the three selection methods, every one of the 30 seeds improved its population-mean fitness over the random generation-0 baseline, and genetic diversity declined smoothly without premature collapse, indicating reliable convergence across seeds (see Fig 4). For the E1 biological metric, the median final correct-settlement rate over seeds was 73–87% across methods, with 28–30 of 30 seeds exceeding a 10% competence threshold (the few non-settling seeds are retained in all reported statistics). This rate is measured on the training configuration itself and is therefore higher than the corresponding validation-battery and head-to-head-baseline rates reported below.

In subsequent simulation rounds, all trained NNs were able to generalize to the new environments, but the degree of quantitative agreement differed markedly across experiments. In E1, correct settlement averaged 54.6±1.6% in V1A (CCA cover 0.41), 45.2±1.5% in V1B (0.20), 61.5±1.6% in V1C (0.60), and 31.7±1.4% in V1D (current on; *n* = 300 runs per setup, 30 genome seeds × 10 RNG seeds; see Table 7). Total settlement, including larvae that settled outside designated reef cells, was higher at 69.0, 60.2, 75.0, and 49.2%, respectively. Correct settlement increased with CCA cover (0.20→0.41→0.60: 45→55→62%; quasibinomial GLM slope on cover β=1.66, *p* < 0.001, *n* = 900 runs) and dropped sharply under an imposed current. Settlement remained spatially aggregated rather than uniform ($R_{CE}=0.16$–0.22). A dedicated patch-size manipulation, in which the maximum tile area was varied at fixed CCA cover, showed a modest, saturating increase in correct settlement with patch size (mean 50.4→50.2→54.6→54.6% for maximum tile areas of 5, 15, 30, and 57 cm^2^; *n* = 300 each; quasibinomial GLM slope on area $\beta =0.0038\;cm^{-2}$, *p* = 0.019).

**Table 7:** E1 settlement and spatial-aggregation results (*n* = 300 runs per setup: 30 genome seeds × 10 RNG seeds), with the original settlement-success values from [33] for comparison. Correct settlement (%) = mean percentage of larvae settling on designated reef cells ± s.e.; total settlement includes larvae settling off reef cells; $R_{CE}$ = Clarke–Evans aggregation index (values <1 indicate aggregation). Original values are the reported per-species settlement success.

	Settlement (%)		$R_{CE}$
	Correct	Total	
V1A (CCA 0.41)	54.6±1.6	69.0	0.20
V1B (CCA 0.20)	45.2±1.5	60.2	0.16
V1C (CCA 0.60)	61.5±1.6	75.0	0.22
V1D (current on)	31.7±1.4	49.2	0.22
*Original settlement success* [33] *(mean ± s.e.):*			
A. valida	16.2±2.1	—	< 1
A. digitifera	33.6±2.4	—	< 1
An. spinosa	47.2±6.6	—	< 1

**Fig 5:**
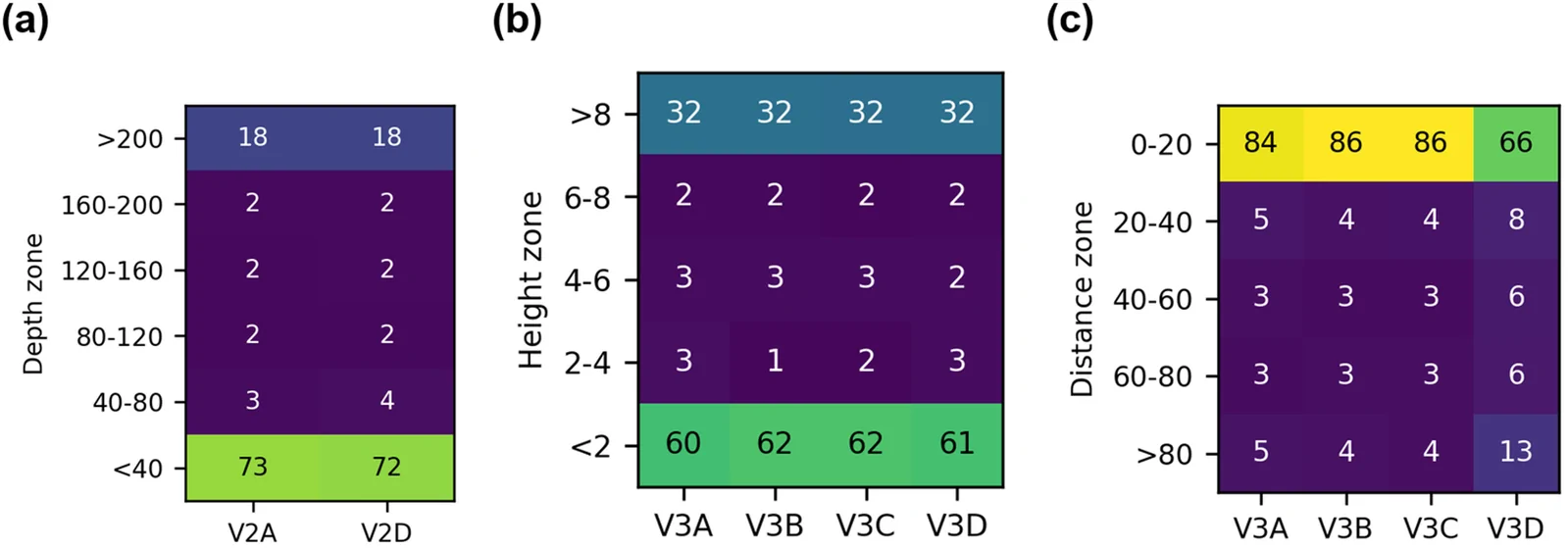
Larval distributions across validation rounds: mean percentage of a run’s larvae in each zone (*n* = 300 runs each, 30 genome seeds × 10 RNG seeds). Cells are annotated with the percentage; colour (viridis) shades the same value on a shared scale. **(a)** E2 vertical distribution across depth zones for the base (V2A) and current (V2D) rounds. Larvae are released near the top of the 220 cm column and descend, so rows run from the surface (> 200 cm, top) down to the tube bottom (< 40 cm, bottom row). The distribution is bimodal, concentrated at the bottom and near the surface with a nearly empty mid-column, and is essentially unchanged under the imposed current (V2D, right column). **(b)** E3 vertical distribution across height zones for validation rounds V3A–V3D. Rows run from the top of the tube (> 8 cm) to the lower tube section (bottom, < 2 cm); the vertical profile shows little variation across the sound scenarios. **(c)** E3 horizontal distribution across distance-to-source zones for validation rounds V3A–V3D. Rows run from the zone nearest the sound source (top) to the farthest (bottom); occupancy concentrates in the near-source zone when sound is present and tracks the source when it is moved (V3B/V3C). (a) E2 vertical. (b) E3 vertical. (c) E3 horizontal.

**Fig 6:**
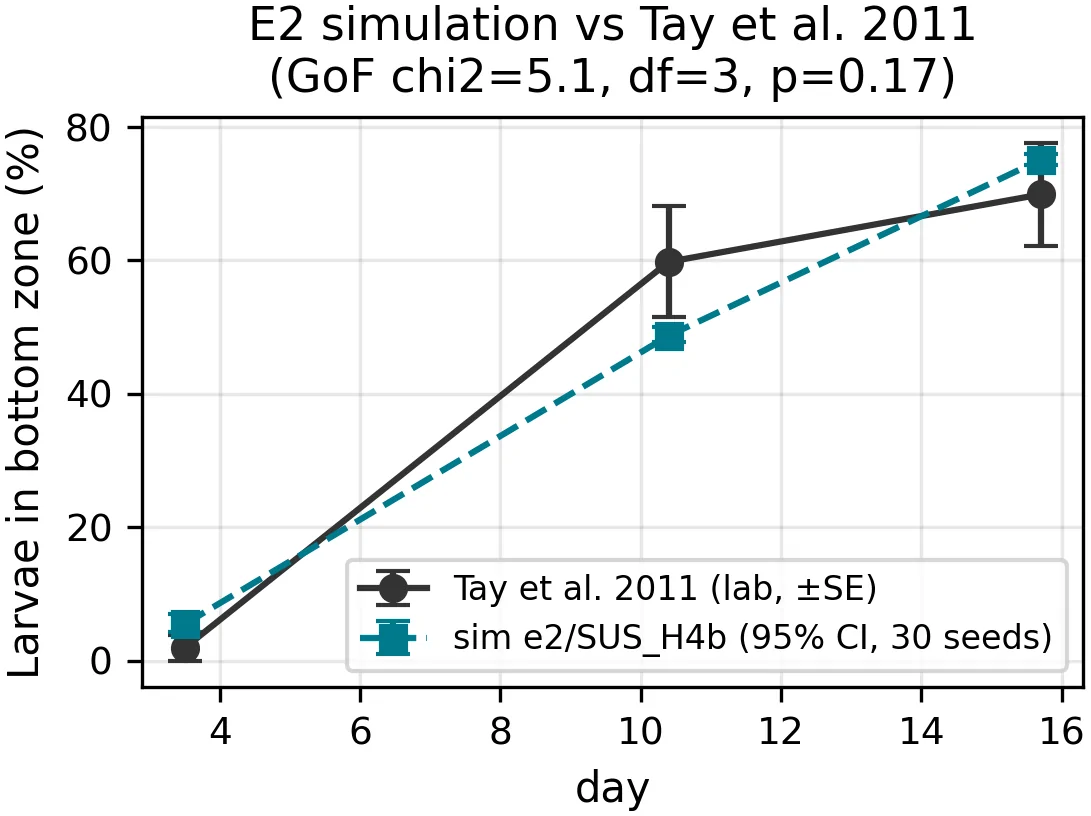
E2 simulation vs. lab depth time course: simulated bottom-zone fraction (with 95% confidence interval) against the values reported by [32] at day 3.5/10.4/15.7.

**Fig 7:**
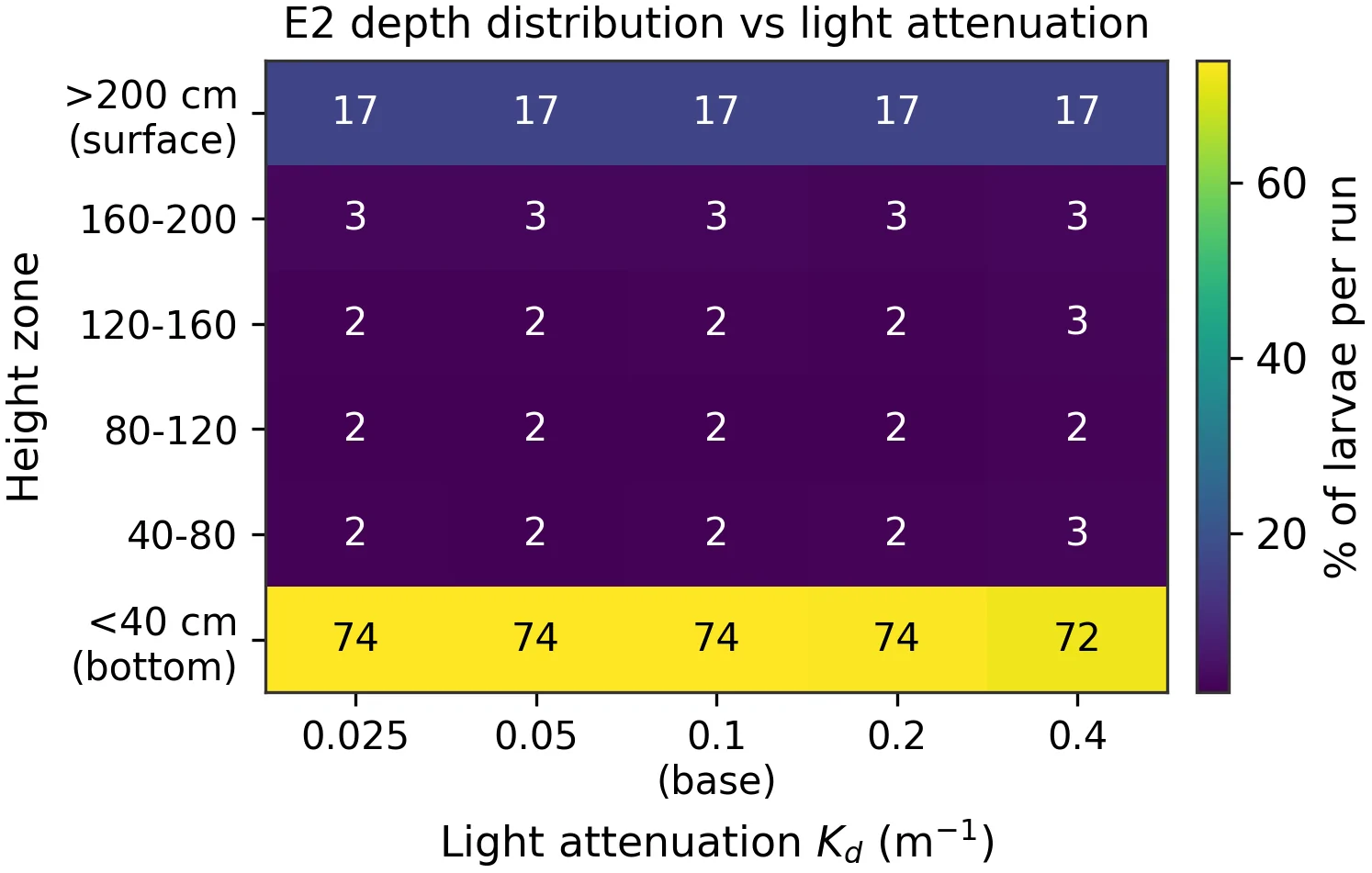
E2 light-attenuation sensitivity. Mean percentage of a run’s larvae in each height zone as the light attenuation coefficient $K_{d}$ is swept over 0.025–$0.4\;m^{-1}$ (base 0.1), with the competency/descent configuration held fixed (10 genome seeds × 3 RNG seeds per level). The bimodal depth profile is essentially invariant across the full range.

In E2, the evolved controllers produced the characteristic *bimodal* vertical distribution rather than a simple accumulation at the bottom: about 73% of larvae ended in the bottom zone and about 18% near the surface, leaving the mid-column nearly empty (*n* = 300; Fig 5a). The bottom-zone fraction rose over time (5.5, 48.7, 75.1% at days 3.5, 10.4, 15.7) toward the laboratory values of [32] (1.9, 59.8, 69.9%; see Fig 6), falling within the reported laboratory standard error at the early and late points, with a mild undershoot at day 10.4. An imposed current (V2D) and a light-attenuation sweep ($K_{d}=0.025$–$0.4\;m^{-1}$, see Fig 7) left the distribution essentially unchanged (72–73% bottom); the current displaced larvae laterally without altering the vertical profile.

For E3, our criterion for successful phonotaxis was that the horizontal occupancy peak tracks the sound source when the source is moved, and becomes diffuse when the sound is removed. Each E3 validation setup was evaluated over 300 runs (30 genome seeds × 10 RNG seeds), as for E1 and E2. With the source held at one end of the tube (V3A), larvae aggregated toward it (mean horizontal position ≈13–21 cm along the 100 cm axis; Fig 5c). When the source was moved to the near (*x* = 15 cm, V3C) and far (*x* = 85 cm, V3B) ends, the occupancy peak followed it: mean final position was ≈21 cm for the near source and ≈79 cm for the far source. At the independent-seed level (per-seed mean *x*, *n* = 30), this shift was significant (paired Wilcoxon signed-rank test, *p* < 0.001; median per-seed shift ≈64 cm). In the no-sound control (V3E) the distribution collapsed to the tube centre (mean ≈50 cm), significantly different from the sound-on placement (paired Wilcoxon signed-rank test, *p* < 0.001). The sound-present distributions were strongly non-uniform across the five distance zones ($\chi^{2}$ vs. uniform, *p* < 0.001, Table 8). A source placed below the tube (V3F) did not induce downward tracking (mean depth essentially unchanged); the E3 response is therefore characterized along the horizontal tube axis, with a vertical profile that varies little across the sound scenarios (Fig 5b).

**Table 8:** E3 horizontal source-tracking and distributional statistics (*n* = 300 per setup: 30 genome seeds × 10 RNG seeds). Mean final horizontal position is along the 0–100 cm tube axis; the source sits at the low-*x* end (V3A), is moved to *x* = 85 (V3B) and *x* = 15 (V3C), is off (V3E), or below the tube (V3F). The horizontal $\chi^{2}$ (vs uniform, five distance zones) and vertical ANOVA (*F*_4,1495_, five height zones) are descriptive non-uniformity measures only (pseudoreplicated; see Methods). Original study: $\chi^{2}=30.5$ (horizontal), *F*_4,25_ = 431.8 (vertical), *p* < 0.0001.

	Mean final *x* (cm)	Horiz. $\chi^{2}$ (df 4)	$F_{4,1495}$ (vert.)	Source
V3A	13.5±1.3	191,040	304.1	low-*x* end
V3B	78.9±1.9	206,857	328.7	moved to *x* = 85
V3C	21.5±1.9	205,123	332.0	moved to *x* = 15
V3D	25.4±2.7	101,831	322.6	low-*x* + current
V3E	50.7±0.5	2,139	297.8	no sound
V3F	50.2±0.2	139,700	280.5	below tube

### 4.1. Robustness to environmental perturbations

**Fig 8:**
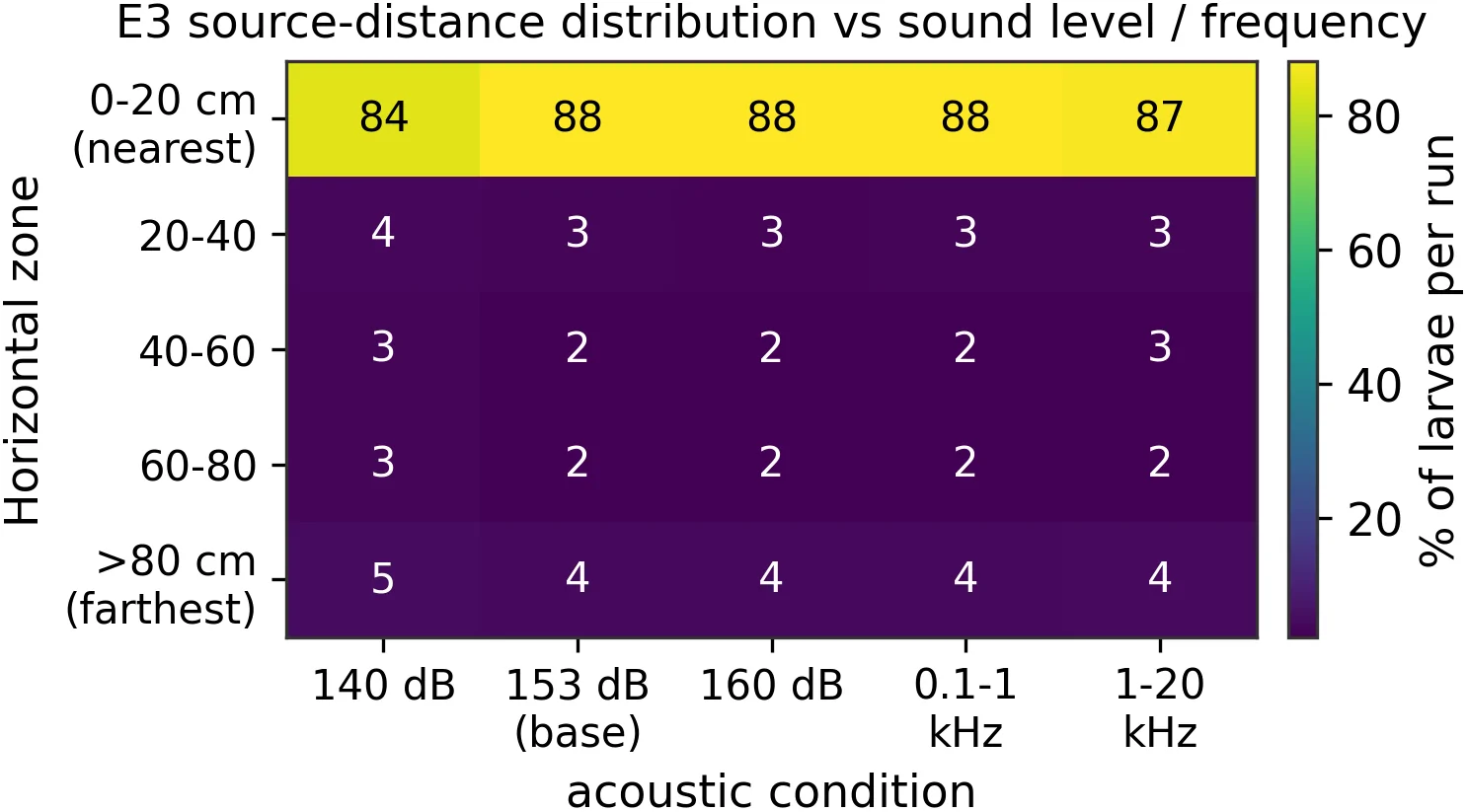
E3 acoustic sensitivity. Mean percentage of a run’s larvae in each source-distance zone across sound source levels (140/153/160 dB) and frequency bands (0.1–1 vs 1–20 kHz), source held at the low-*x* end (10 genome seeds × 3 RNG seeds per condition). Source-directed aggregation strengthens with source level and is largely insensitive to the frequency band, consistent with the reduced scalar particle-motion representation.

Across the perturbation runs, the three experiments responded differently. In E1, correct settlement rose with CCA cover (45/55/62% at cover 0.20/0.41/0.60). In E2, the vertical distribution was essentially invariant to the light-attenuation sweep ($K_{d}=0.025$–$0.4\;m^{-1}$; 72–73% bottom throughout, see Fig 7). In E3, source tracking persisted when the source was moved but was lost without sound, and degraded under sensory noise. Source-directed aggregation strengthened with source level (nearest-zone occupancy 84% at 140 dB, rising to and saturating at 88% over 153–160 dB) and was largely insensitive to the frequency band (see Fig 8). The imposed current (V1D/V2D/V3D) had the largest effect in E1, where correct settlement fell to 32%; in E2 it left the vertical distribution nearly unchanged; and in E3 it shifted the occupancy peak modestly away from the source (mean *x* from ≈14 to ≈25 cm) while preserving source-directed aggregation.

### 4.2. Controller analysis and robustness

Beyond the primary validation, we compared the evolved controllers against simpler baselines and analyzed their internal structure and robustness, using the final controller sets.

**Simpler-controller baselines.** In a dedicated head-to-head comparison, we evaluated each evolved controller against two references on the same primary scenario (so the values below are not identical to the validation-battery means of Tables 7 and 8): an untrained, random genome and a fixed reactive rule, specifically a gradient taxis along the directional cue with a settlement threshold. In E1, the evolved controllers settled correctly far more often than either baseline (median 44.9% vs. 0.8% random and 7.5% reactive). In E3, only the evolved controllers oriented toward the source (mean distance-zone position ≈22 of 100 cm, vs. the ≈50 cm tube centre for both baselines). In E2, the reactive rule performed comparably to the evolved network (84% vs. 75% of larvae in the lowest zone), and both exceeded the random controller (39%).

**Fig 9:**
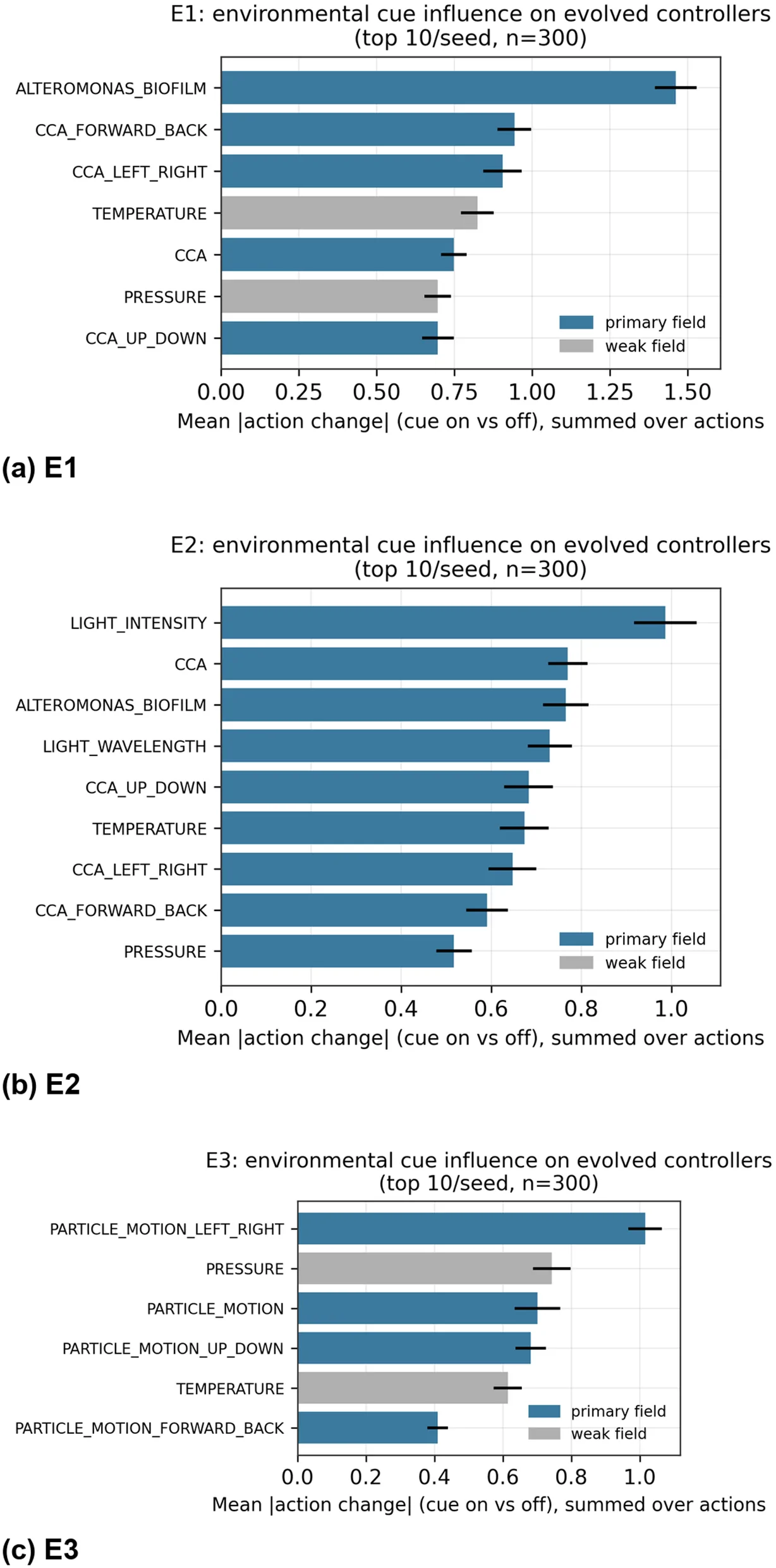
Cue dominance in the evolved controllers: the influence of each sensory channel, measured as the mean change in the controller’s action when that cue is toggled on versus off (controller-intrinsic sensitivity) and cross-checked against the realized, variance-weighted influence computed from the recorded sensor traces. Bars are coloured by whether the field is the primary manipulated cue or a weakly varying one. E1 is dominated by the CCA and biofilm channels, E3 by particle motion, and E2 shows only weak cue influence. (a) E1. (b) E2. (c) E3.

**Cue dominance.** Feeding the recorded per-step sensor traces of the final controllers back through their networks to obtain a variance-weighted realized influence, complemented by synthetic single-cue sweeps measuring controller-intrinsic sensitivity, gave a consistent picture: E1 decisions were dominated by the CCA and *Alteromonas* biofilm channels, E3 by the directional particle-motion channel, and E2 showed only weak realized cue influence (see Fig 9).

**Fig 10:**
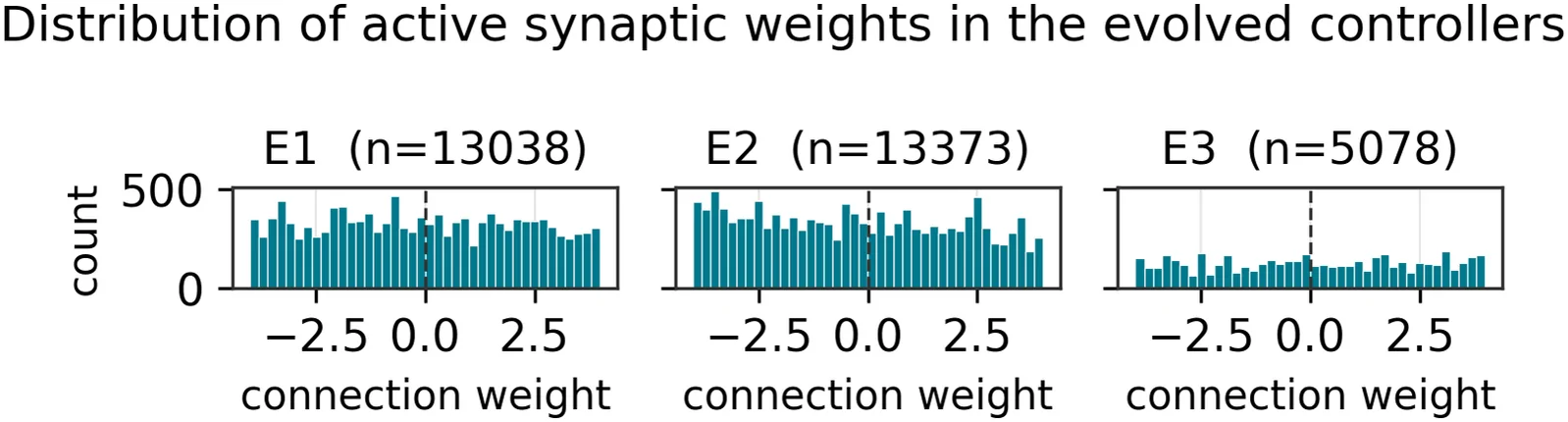
Distribution of the active, post-pruning synaptic weights of the evolved controllers, pooled over the top-10 elite genomes of all 30 seeds per experiment. Weights are broadly distributed and approximately zero-centred, with a near-balanced split of excitatory (positive-weight) and inhibitory (negative-weight) connections; the dashed line marks zero. Cue selectivity is not reflected in the raw weight magnitudes, which are comparable across sensory-channel classes, but emerges from network topology and realized sensory variation, as quantified by the cue-dominance analysis (Fig 9).

**Weight distribution.** The evolved controllers were characterized structurally by pooling, for the top-10 elite controllers across all 30 seeds of each final set, the connection weights that survived genome pruning (see Fig 10). After pruning, the effective controllers retained on average 10–11 active hidden neurons and 46–71 weighted connections per genome, with both direct sensor-to-action and neuron-mediated pathways present in all three experiment groups, and the most frequent connections differed between E1, E2, and E3. In all three experiments the weights were broadly distributed and centred near zero (E1 mean −0.08, E2 −0.26, E3 + 0.08; s.d. ≈2.3), with an approximately balanced split of excitatory and inhibitory connections (48/52%, 46/54%, 50/50% for E1/E2/E3). Notably, the mean absolute weight of the incoming connections was similar across sensory-channel classes (≈1.6–2.2): cue selectivity therefore does not arise from systematically larger weights on the “relevant” sensors, but from network topology and from which sensory fields actually vary in a given geometry.

**Fig 11:**
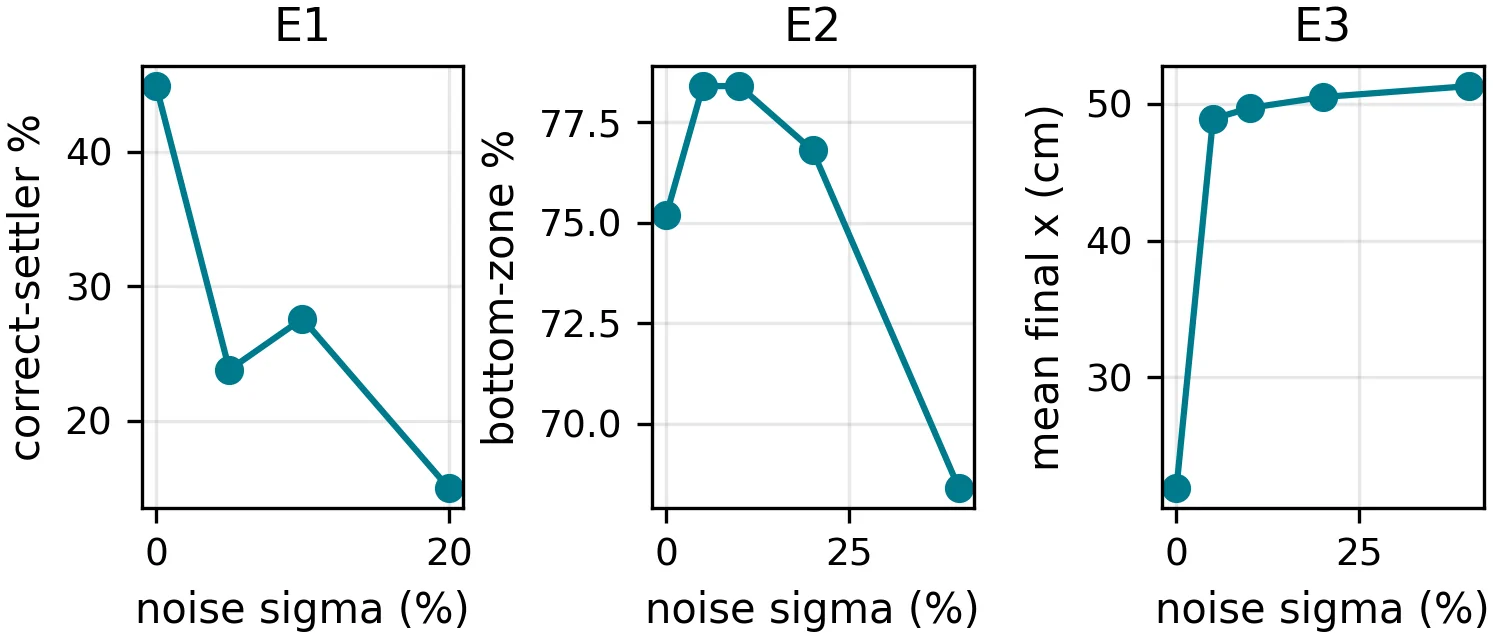
Sensory-noise robustness: the primary metric of each experiment versus additive Gaussian sensor noise (standard deviation as a fraction of the sensor range). E2 is essentially flat, E1 degrades gracefully (breakpoint ≈10–20%), and E3 phonotaxis collapses at ≈5%.

**Sensory-noise robustness.** Adding Gaussian noise (standard deviation 5–40% of the sensor range) to the trained controllers at validation revealed markedly different sensitivities (see Fig 11). E2 was essentially unaffected up to 40% noise. E1 degraded gradually, with correct settlement roughly halving between 5 and 10% noise and a breakpoint near 10–20%. E3 phonotaxis was the most fragile: source tracking collapsed to the tube centre at only 5% noise.

## 5. Discussion

Overall, the model reproduced cue-directed behavior in all three experiments, but the strength of quantitative agreement varied across experiments. Table 9 summarizes, for each experiment, the simulated outcome, the laboratory reference, and the main remaining mismatch.

**Table 9:** Overview of agreement and mismatch between the laboratory studies and the simulation outcomes.

	Simulation Result	Laboratory Reference	Main Mismatch
**E1**	Correct settlement rises monotonically with CCA cover: 45.2/54.6/61.5% at cover 0.20/0.41/0.60 (V1B/V1A/V1C, *n* = 300 each), falling to 31.7% under current (V1D); patterns aggregated ($R_{CE}=0.16$–0.22).	Original settlement success was 16.2/33.6/47.2% for *A. valida*/*A. digitifera*/*An. spinosa*, with aggregated patterns.	The CCA-driven direction matches the laboratory finding; absolute settlement is of the same order but somewhat higher than the lab species range (an over-settling tendency in the abstracted single-tile environment).
**E2**	The population ended bimodal: ≈73% bottom, ≈18% surface, empty mid-column; bottom-fraction 5.5/48.7/75.1% at day 3.5/10.4/15.7.	Tay reported bottom fractions of 1.9/59.8/69.9% at day 3.5/10.4/15.7, bimodal with a near-empty mid-column.	The simulated fraction lies within Tay’s reported s.e. at day 3.5 and 15.7; a mild undershoot at day 10.4 (gradual vs. steep rise).
**E3**	The occupancy peak tracks the moved source (mean position ≈21→79 cm as the source moves from *x* = 15 to *x* = 85 cm; median shift ≈64 cm) and collapses to the tank centre (≈50 cm) with no sound.	The original experiment reported source-directed aggregation (horizontal $\chi^{2}=30.5$, *p* < 0.0001) that vanished in the silent control.	Directional phonotaxis is reproduced; the source-tracking (moving-source) test is used rather than $\chi^{2}$-vs-uniform, because tube-end accumulation makes even the silent distribution mildly non-uniform.

**E1: settlement on CCA.** Correct settlement increased monotonically with CCA cover and showed a modest positive patch-size effect, consistent with the literature identifying CCA as a primary settlement cue [35]. The simulated percentages are of the same order as the laboratory values but somewhat higher than the per-species range, i.e., an over-settling tendency in the abstracted single-tile environment rather than a failure of cue-directed settlement. Two caveats concern spatial detail rather than the presence of this. First, larger tiles increase the total CCA-covered area, so patch size is partly confounded with total cue availability; the controlled maximum-tile-area sweep therefore isolates the size effect more cleanly than an observational area regression. Second, the direct larva–larva contact aggregation reported by [33] was not reproduced, because the model has no larva–larva sensing: once a larva reaches a reef cell, settlement is the terminal goal state, and larvae cannot perceive one another or any post-settlement signal. This has been a deliberate simplification, as there is little evidence that swimming larvae detect one another, although freshly settled larvae may release settlement cues [14], a mechanism left for future work [33]. further noted that small residual water movements in their aquaria, not modeled here, may also have shaped the observed spatial relationship.

**E2: vertical distribution.** The simulated bottom-zone fraction matched the laboratory values within the reported standard error at the early and late time points and reproduced the characteristic bimodal top/bottom split with a near-empty mid-column [32], an emergent shape that was not itself a calibration target. The one systematic deviation, a mild undershoot at the middle time point, follows directly from drawing each larva’s competency age from a uniform interval (yielding near-linear rather than sigmoidal accumulation). A clustered onset distribution would sharpen the transition. Because the distribution was invariant to the chemical and light cues and to an imposed current, and because a fixed reactive rule reproduced it as well as the evolved network, the E2 depth profile is best interpreted as competency-gated geotaxis rather than as an evolved cue-integration behavior. E2 thus serves as a boundary case delimiting where an evolved controller is required: depth arises from a simple maturation-gated mechanism, whereas the settlement of E1 and the phonotaxis of E3 genuinely demand evolution.

**E3: phonotaxis.** The simulated aggregation toward the sound source was stronger than in the laboratory but qualitatively consistent with the original non-random, source-directed response. Its weaker sensitivity to fine spectral detail reflects the simplified sound field: a reduced scalar particle-motion proxy in an idealized geometry that omits reflections, boundary effects, and flow-acoustics interactions. The resulting shallow gradient (≈3%/m) also underlies the fragility of E3 phonotaxis under sensory noise (tracking collapsed at only 5% noise): a weak directional signal is easily swamped, a biologically meaningful limit on cue-guided orientation at this scale.

**Controller complexity.** Across the three tasks, the selection-method and hyperparameter analyses indicate that the appropriate controller complexity depended on the behavior being learned rather than being uniformly “more is better”. Reducing the genome length lowered E1 settlement, which needs the full controller complexity to locate and settle on the reef, but improved E3 phonotaxis, where a smaller controller tracked the source more reliably; a reduced population gave the best E2 depth match. The final configurations therefore differed by task: E1 used the baseline, E3 a reduced genome, and E2 a reduced population. More broadly, the agents evolved from random, exploratory behavior to purposeful, cue-directed action, confirming that NE recovered the intended behaviors.

**Interpretability.** Beyond behavior, the structure of the evolved controllers (see Fig 10) bears on interpretability. That the most frequent connections differed between E1, E2, and E3 indicates that the controllers did not collapse to one trivial wiring pattern; the structural summaries alone, however, do not establish which cues dominate online decisions, which the cue-dominance analysis addresses. The explanatory value of the NE approach is therefore limited but concrete: it exposes a learnable, inspectable sensor-to-action mapping that transfers across modified cue environments.

**Limitations and future work.** Some discrepancies stem from deliberate simplifications such as reduced hydrodynamics, idealized cue fields, uniform reef-cell representation, and the absence of larva–larva or post-settlement social cues, while others reflect genuine uncertainty about how larvae integrate multiple sensory modalities under changing conditions. Greater realism, e.g., more detailed reef structure, refined parameter distributions, explicit hydrodynamic variability, and additional cue sensing such as interspecies or post-settlement signals, together with combining the three individually tested cues into a single multi-cue experiment, are natural next steps. The reproduced behavior likely reflects a mix of real larval tendencies and artefacts of the model’s abstraction (fitness shaping, simplified geometry and cue fields), and disentangling the two remains an open problem.

## 6. Conclusion

In summary, this paper presented a NE-driven ABM which reproduced key larval responses to CCA, depth, and sound under controlled conditions and generalized to altered setups. As discussed, realism limits remain, suggesting next steps in environmental detail and scalability. Yet, our experiments indicate that incorporating NE into an ABM enables the simulation of adaptive sensory-driven larval behaviors, a feature that extends beyond the fixed deterministic processes of many conventional mechanistic models. By allowing agents to learn and adjust their actions in response to dynamic environmental cues, our model captures aspects of coral larval settlement that remain underrepresented in existing frameworks. The ability of evolved networks to generalize to novel conditions is a promising indicator of their potential to better reflect the complexities of larval dispersal and settlement in real reef environments. Such models in turn can inform real-world measures to increase larval settlement success, and ultimately the rebuilding of coral cover. However, it is important to recognize that the controlled experimental conditions employed here do not fully encompass the variability of natural systems. More empirical research and model refinements are required to assess the broader applicability of this approach to inform effective reef restoration and management strategies.
